# Neonatal cerebral imaging: feasibility and image quality using an MR-compatible incubator equipped with a 16-channel head coil

**DOI:** 10.1007/s00330-025-12219-3

**Published:** 2025-12-22

**Authors:** Patricia Tischendorf, Maja Wiesmann, Heymut Omran, Tobias Krähling, Julia Sandkötter, Walter Heindel, Laura Beck

**Affiliations:** 1 Clinic for Radiology, University of Münster and University Hospital Muenster, Münster, Germany; 2 Department of General Pediatrics, University Children’s Hospital Muenster, Münster, Germany

**Keywords:** Magnetic resonance imaging, Infant, Premature birth, Brain, Anesthesia

## Abstract

**Objectives:**

To address the needs of preterm and term neonates undergoing cerebral magnetic resonance imaging (cMRI), using a recently developed MR-compatible incubator (icMRI) equipped with a 16-channel receiver head coil was evaluated for feasibility and image quality compared to conventional cMRI (ccMRI) using a 16-channel adult head coil.

**Materials and methods:**

One hundred three consecutive neonates (46 females; 40 ± 3 gestational weeks) underwent clinically indicated cMRI, 53 were examined with an incubator, and 50 without. Normally distributed variables of the two groups were compared using the Student’s *t*-test. The Wilcoxon signed-rank test was applied for unequal variances. Image quality was subjectively rated on a 5-point scale by two pediatric radiologists, with interobserver agreement and signal-to-noise ratios in the parieto-occipital white matter (SNRc) and basal ganglia (SNRb) calculated. Interobserver agreement was assessed using the quadratic weighted Cohen’s kappa test (ϰ). Mean SNR values between groups were compared using the Student’s *t*-test.

**Results:**

Neonates who underwent icMRI had a significantly lower weight at imaging (*p* = 0.04). Total imaging time was significantly shorter (*p* = 0.03) using an incubator. icMRI led to a significant increase in SNRc and SNRb across all sequences (*p* ≤ 0.006), while diagnostic image quality improved from 3.5 to 4.3 points, and image motion artifacts decreased from 31% to 14% with substantial to almost perfect interobserver agreement. Furthermore, icMRI was associated with reduced need for general anesthesia (*p* = 0.03).

**Conclusion:**

icMRI is correlated with better feasibility, improved image quality, shorter scan times, and reduced need for general anesthesia compared to ccMRI.

**Key Points:**

***Question****Neonatal cerebral MRI requires a precisely controlled environment, including optimal temperature, humidity, and real-time vital monitoring to ensure safety and stability during imaging*.

***Findings*** *A recently developed icMRI with an integrated 16-channel radiofrequency receiving head coil is associated with improved feasibility and image quality in neonatal cMRI*.

***Clinical relevance*** *Newborns benefit from a recently developed icMRI with an integrated 16-channel head coil that optimizes safety, handling, monitoring, and transport, while also correlating with improved image quality, reduced scan time’s and reduced need for general anesthesia*.

**Graphical Abstract:**

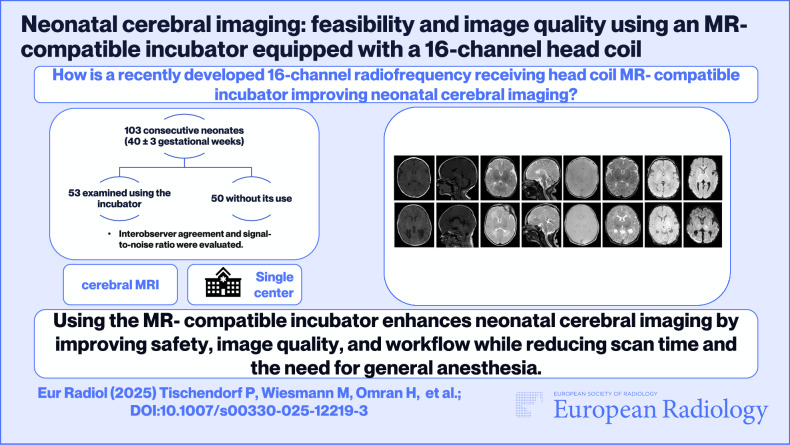

## Introduction

Cerebral magnetic resonance imaging (cMRI) of neonates poses considerable challenges due to the complexities of patient transport, monitoring requirements, and the inherent difficulty of minimizing patient motion, which can compromise image quality. Despite these hurdles, the critical role of cMRI in assessing both preterm and term neonates is well established, given its unparalleled ability to detect ischemic changes, diffuse white matter injuries, and cerebellar abnormalities [1–4]. Both term and particularly preterm neonates require meticulously controlled environmental conditions to ensure their stability during imaging. Key considerations include thermoregulation, continuous hemodynamic monitoring, and, in many cases, ventilatory support [5]. To counteract motion artifacts, sedation of neonates might be deemed necessary, also demanding continuous monitoring throughout the examination [6]. The introduction of MR-compatible incubators has revolutionized neonatal imaging by enabling safer evaluations of preterm and critically ill term infants. Previous studies have highlighted the safety of these systems, alongside the excellent image quality and additional diagnostic insights they provide, even in small patient cohorts [7–10]. Nonetheless, limited data exist comparing the image quality obtained using integrated incubator-coil systems to that of conventional MR equipment [7–9, 11]. A recently developed MR-compatible incubator system integrates a 16-channel radiofrequency receiving head coil. It replaces the previous 8-channel version and offers improved patient access, reduced noise exposure, a wider lying surface, and a lighter overall design. The system maintains shielding for neonates during transport and imaging while regulating the temperature and humidity within the patient environment. Other MR-compatible incubator systems are primarily designed for safe transport and have limited imaging-related features because they lack full incubator functionality and integrated multi-channel coils. Previous studies evaluating the image quality of MR-compatible incubators have used systems with 8-channel head coils or no integrated coils [7–13]. Currently, no imaging data is available on this recently developed system with a 16-channel radiofrequency receive head coil, which served as an additional motivation for our study. Therefore, our study aimed to evaluate this recently developed incubator in terms of the feasibility and image quality across various sequences in both term and preterm neonates compared to previous conventional cMRI (ccMRI) examinations using a 16-channel radiofrequency adult head coil.

## Methods and materials

### Study population

This study was approved by the local ethics committee. We conducted a retrospective review of clinically indicated cerebral MR examinations in a consecutive cohort of 103 term and preterm neonates. These examinations were conducted over a period from May 2018 to September 2022 without using the incubator, followed by an additional period from November 2022 to November 2024 with the use of the incubator. The patients were categorized into two groups depending on whether the incubator was used (icMRI) or not. There were no exclusion criteria for the icMRI group. All consecutive examined neonates who received a cMRI with the incubator were included. In the ccMRI group, among all the neonates who underwent cMRI with the conventional 16-channel radiofrequency adult head coil, the ones who met the size requirements for the MR-compatible incubator for comparability purposes with the icMRI group were included. In addition, various clinical parameters, including heart rate, oxygen saturation, and temperature, as well as the need for sedation or general anesthesia, were retrieved from the clinical records and documented. Within the icMRI group, the patient cohort was further analyzed based on the type of MRI device utilized.

### MR—compatible incubator

**Fig. 1:**
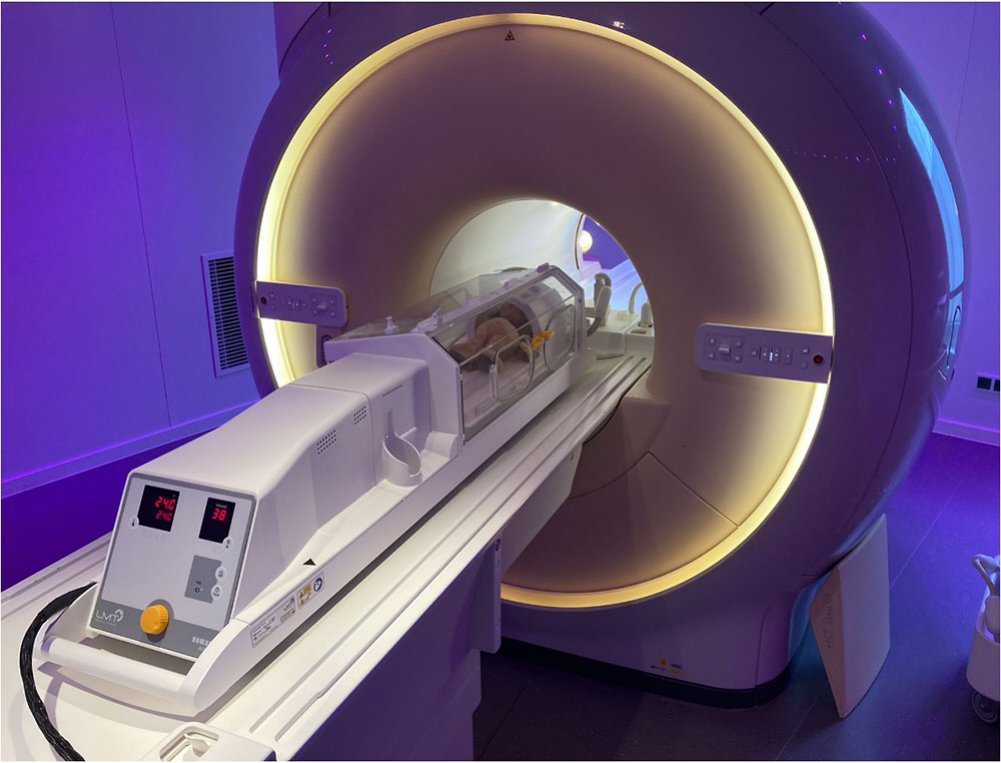
Picture of the recently developed 16-channel radiofrequency receiving head coil, MR-compatible incubator

The utilized system was the LMT nomag®IC Advanced 1.5 MR Incubator System (Lammers Medical Technology), pictured in Fig. 1. This system encompasses an incubator, a non-magnetic trolley and power supply for transportation, a dedicated 16-channel receiving head coil (LMT Neonatal Head Coil 1.5-T; Lammers Medical Technology), and a coil connector adapter for connection to the Philips dStream interface. Designed for neonates and infants up to 4.5 kg in weight and 55 cm in length, the incubator ensures air temperature control (28–39 °C; 82.4–102.2 °F) and humidity control (30–70%).

### Handling

The preparation for MRI was similar in both groups. Noise reduction was achieved using Mini Muffs (Natus Medical Incorporated). Throughout the examination, continuous monitoring of heart rate and oxygen saturation utilizing an Expression MR400 patient monitor system (Philips Healthcare) took place. Temperature measurement was conducted before the MRI and on the ward. The recorded measurements were documented in an observation protocol. Pediatricians and nurses with specialized MRI training oversaw patient monitoring.

Patients scanned within the incubator were initially positioned in the device at the neonatal intensive care unit before being transported to the MRI unit. Neonates who underwent scanning without the incubator were positioned in the 16-channel radiofrequency adult head coil directly in the MRI suite. There were no adverse clinical events relating to the transport of the infant or the scan itself in either of the two groups.

### MR examinations

MRI was performed in the ccMRI group on a 1.5-T Ingenia (Philips Healthcare) and in the icMRI group on a 1.5-T Achieva SmartPath to dStream (Philips Healthcare) and also on a 1.5-T Ingenia (Philips Healthcare). The 1.5-T Ingenia and the 1.5-T Achieva SmartPath to dStream are comparable devices, differing in the bore diameter, which is 70 cm and 60 cm, respectively. All sequence parameters, including slice thickness, repetition time, echo time, flip angle, slice distance, field-of-view, and recon pixel size, were recorded in the DICOM (digital imaging and communications in medicine) metadata dictionary for every sequence (Table 1). The imaging protocols used included axial Dual TSE, axial and sagittal T1-weighted spin echo imaging, axial and sagittal T2-weighted spin echo imaging, axial susceptibility weighted imaging, and axial diffusion-weighted sequences. Depending on the indication for the examination, the required sequences were used. The DICOM data files for each MRI examination were reviewed, and the start time of the initial localizer sequence, as well as the end time of the last acquired sequence, were documented, whereby the difference between these two time stamps was used as the examination time.

**Table 1:** MRI sequence parameters for both groups

MRI parameters icMRI group	T1 transversal	T1 sagittal	T2 transversal	T2 sagittal	Dual TSE 1	Dual TSE 2	SWI	DWI
Recon pixel size (mm)	0.4 × 0.4	0.4 × 0.4	0.4 × 0.4	0.3 × 0.3	0.4 × 0.4	0.4 × 0.4	0.5 × 0.5	1.7 × 1.7
Field of view (cm)	160	160	150	160	150	150	150	150
Repetition time-/echo time (ms)	25/5	25/5	5396/68	4500/140	4500/15	4500/140	52/12	3224/88
Flip angle (°)	30	30	90	90	90	90	20	90
Number of averages	1	1	1	2	1	1	1	1
Slice thickness (mm)	1	1	3	3	3	3	2.5	4.4
Spacing between slices (mm)	1	1	4	3.3	3.3	3.3	1.2	4
MRI parameters ccMRI group	T1 transversal	T1 sagittal	T2 transversal	T2 sagittal	Dual TSE 1	Dual TSE 2	SWI	DWI
Recon pixel size (mm)	0.25 × 0.25	0.6 × 0.6	0.4 × 0.4	0.3 × 0.3	0.4 × 0.4	0.4 × 0.4	0.6 × 0.6	1.3 × 1.3
Field of view (cm)	256	256	140	180	230	230	230	230
Repetition time-/echo time (ms)	25/5	25/5	7243/120	3773/120	4500/13	4500/120	52/12	3196/94
Flip angle (°)	30	30	90	90	90	90	20	90
Number of averages	1	1	1	2	2	2	1	1
Slice thickness (mm)	1	1	3	3	5	5	2.5	5
Spacing between slices (mm)	1	1	3.3	3.3	5.5	5.5	1.2	6

*cMRI* cerebral magnetic resonance imaging, *icMRI group* patient group undergoing cMRI with a recently developed MR-compatible incubator, *ccMRI group* patient group undergoing conventional cMRI, *DWI* diffusion-weighted imaging, *SWI* susceptibility-weighted imaging, *TSE* Turbo spin echo

### Image quality data

Two pediatric radiologists with 11 and 13 years of experience (P.T. and L.B.) assessed image quality both subjectively and objectively while remaining blinded to patient details and scanning methods (conventional equipment or MR-compatible incubator). Subjective image quality was estimated according to a 5-point Likert scale based on image sharpness and clarity of the grey–white matter interfaces, evaluating overall [13]. Additionally, the presence or absence of motion artifacts in the examination sequences was documented, along with any pathological findings detected. The signal-to-noise ratio (SNR) was quantified for every sequence of each MR examination, involving the placement of a region of interest ca. 10 mm^2^ over a constant point in the parieto-occipital white matter (SNRc) and in the basal ganglia (SNRb), and determining the mean signal intensity. SNR was calculated as: SNR = mean signal of tissue/standard deviation of background noise [14].

### Statistical methods

Data acquisition was performed in Microsoft Excel (Microsoft Corporation), and descriptive statistical measures were calculated. To evaluate subjective image quality, interobserver agreement between the two readers was analyzed using the quadratic weighted Cohen’s kappa test (ϰ). The interpretation of ϰ values was as follows: ϰ < 0.20, slight agreement; ϰ = 0.21–0.40, fair agreement; ϰ = 0.41–0.60, moderate agreement; ϰ = 0.61–0.80, substantial agreement; and ϰ = 0.81–1.0, almost perfect agreement. To evaluate the normality of data distribution, the Kolmogorov-Smirnov test was applied. Normally distributed data were analyzed using the Student’s *t*-test, while the Wilcoxon signed-rank test was used in cases of unequal variances. A *p*-value < 0.05 was considered statistically significant. The independent samples Student’s *t*-test was employed to compare mean SNR values between the two groups. All statistical analyses were performed using IBM SPSS Statistics version 29.0 (IBM Corporation). Data were pseudo-anonymized and processed in compliance with local data protection regulations.

### Anesthetic protocols

Airway management decisions were made by the involved pediatricians and an anesthesiologist based on patient-specific factors. Our pediatricians perform sedation with chloral hydrate and melatonin to allow the children to breathe spontaneously. For induction of general anesthesia, sufentanil in combination with propofol was administered, while maintenance was achieved using sevoflurane.

## Results

### Patients’ characteristics

103 neonates (46 females; mean gestational age at examination 40.5 ± 3.2) comprised the study population. The most common imaging reason was asphyxia (25/103) and further brain clarification (25/103), followed by intracranial hemorrhage (23/103) and by brain anomaly (23/103), with no significant difference between the groups (Table 2). The average gestational age was 37.4 ± 3.6 weeks, with a mean birth weight of the neonates was 2900 ± 800 g. Neonates examined using the incubator had significantly lower weights at both birth and the time of examination (*p* = 0.005; *p* = 0.04, Table 2).

**Table 2:** Patients characteristics and MRI procedure of both groups

	All	icMRI group	ccMRI group	*p*
Patient’s characteristics				
Female	46	22	24	0.5
Gestation week birth	37.4 ± 3.6	37 ± 4.3	37.9 ± 2.6	0.6
Gestation week examination	40.5 ± 3.2	40.5 ± 3.0	40.6 ± 3.3	0.4
Height at birth (cm)	48.2 ± 5.4	46.8 ± 6.3	49.8 ± 3.6	**0.01**
Weight at birth (grams)	2900 ± 800	2600 ± 900	3200 ± 700	**0.005**
Head circumference at birth (cm)	34 ± 3	33 ± 4	34.0 ± 2.7	0.6
Height at examination (cm)	50 ± 4	50 ± 4	51 ± 5	0.1
Weight at examination (grams)	3300 ± 800	3100 ± 600	3400 ± 900	**0.04**
Head circumference at examination (cm)	34.6 ± 2.8	34.4 ± 0.4	34.8 ± 0.5	0.3
Indication				
Stroke	7	4	3	0.8
Intracranial hemorrhage	23	11	12	0.7
Asphyxia	25	16	9	0.2
Brain anomaly	23	8	15	0.1
Further brain clarification	25	14	11	0.6
Anesthetic procedure				
General anesthesia	13	3	10	**0.03**
Sedation	87	47	40	0.3
Feed and sleep	3	3	0	0.1
Vital parameters				
Temperature before MRI (°C)	36.9 ± 0.4	36.9 ± 0.3	36.8 ± 0.5	0.8
Temperature after MRI (°C)	36.7 ± 0.5	36.8 ± 0.4	36.7 ± 0.6	0.1
Heart rate (bpm)	133 ± 18	134 ± 19	132 ± 18	0.5
Oxygenation (%)	98.0 ± 1.8	98.0 ± 1.7	98.0 ± 1.8	0.9
MRI-examination				
Imaging time (mins)	28 ± 15	25 ± 14	30 ± 15	**0.03**
T1 transversal (# of applications)	78	39	39	0.6
T1 sagittal (# of applications)	94	45	49	0.2
T2 transversal (# of applications)	28	15	13	0.8
T2 sagittal (# of applications)	94	50	44	0.2
Dual TSE (# of applications)	83	42	41	0.7
SWI (# of applications)	90	46	44	0.9
DWI (# of applications)	97	47	50	**0.02**

*cMRI* cerebral magnetic resonance imaging, *icMRI group* patient group undergoing cMRI with a recently developed MR-compatible incubator, *ccMRI group* patient group undergoing conventional cMRI, *DWI* diffusion-weighted imaging, *SWI* susceptibility-weighted imaging, *TSE* Turbo spin echo

Statistically significant values are shown in bold

### MRI examination and image quality

The mean examination time was significantly lower with the incubator compared to the group without it (25 ± 14 min vs 30 ± 15 min; *p* = 0.03). The only differences in the number of applied sequences between the two groups were observed in diffusion weighted imaging (DWI), favoring the icMRI group, as shown in Table 2. For all other sequences, there was no significant difference in the number of applied sequences between the two groups.

**Fig. 2:**
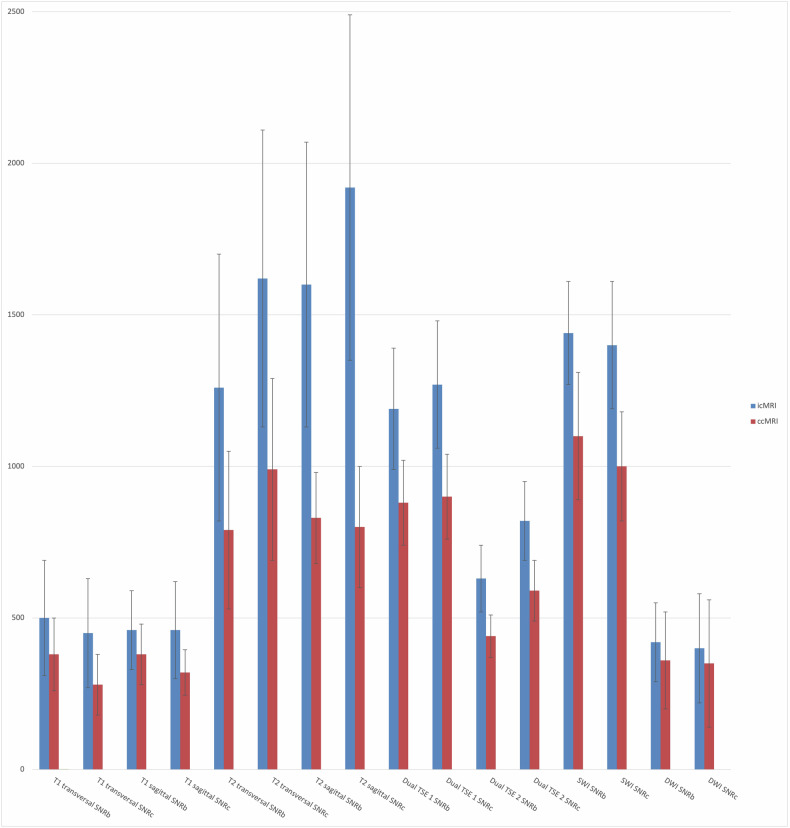
Average data with standard deviation of signal-to-noise ratios in the parieto-occipital white matter (SNRc) and SNRb across all sequences comparing the icMRI and ccMRI groups. cMRI, cerebral magnetic resonance imaging; icMRI, cerebral MRI using a recently developed MR-compatible incubator; ccMRI, conventional cerebral magnetic resonance imaging; Dual TSE 1 and 2, dual turbo spin echo in T1 and T2 weighting; DWI, diffusion-weighted imaging; SWI, susceptibility-weighted imaging; SNRb, Signal-to-noise ratios in the basal ganglia; TSE, Turbo spin echo

**Fig. 3:**
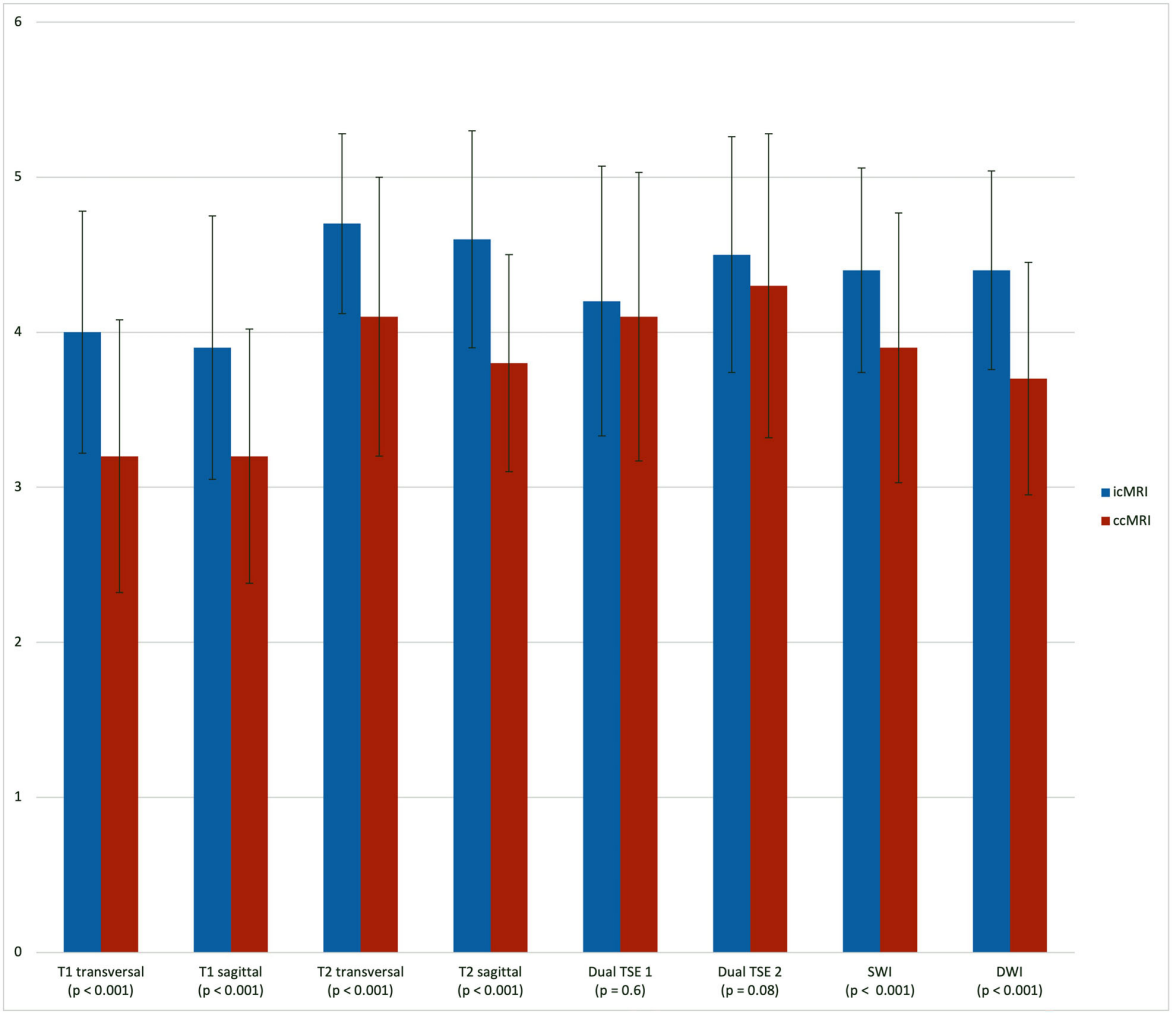
Average data with standard deviation of overall subjective diagnostic image quality for both groups across all sequences. cMRI, cerebral magnetic resonance imaging; icMRI, cerebral MRI using a recently developed MR-compatible incubator; ccMRI, conventional cerebral magnetic resonance imaging; Dual TSE 1 and 2, dual turbo spin echo in T1 and T2 weighting; DWI, diffusion-weighted imaging; SWI, susceptibility-weighted imaging; TSE, Turbo spin echo

**Fig. 4:**
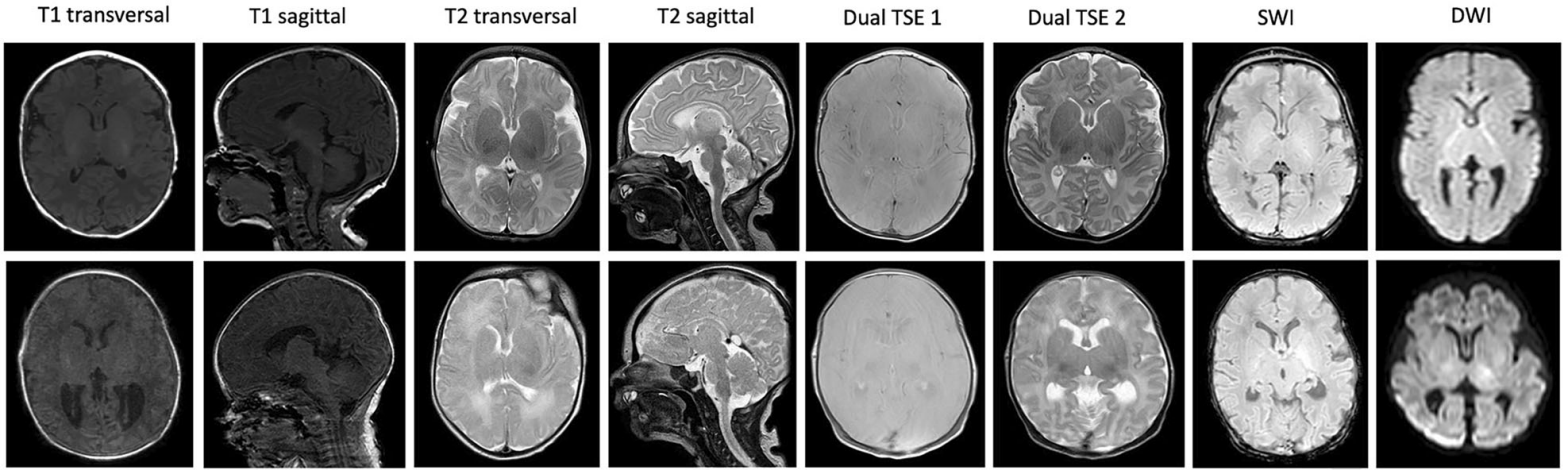
An overview of all applied sequences is presented in the upper row using the recently developed 16-channel radiofrequency receiving head coil MR-compatible incubator and in the lower row without it. From left to right, the sequences include T1 transversal, T1 sagittal, T2 transversal, T2 sagittal, Dual TSE 1 and 2, dual turbo spin echo in both T1 and T2 weighting, as well as the SWI sequence and the diffusion-weighted imaging (DWI) sequence; TSE, Turbo spin echo; SWI, susceptibility-weighted imaging

Using the incubator, SNRc and SNRb were significantly higher across all sequences (*p* ≤ 0.006), compared to ccMRI examination (Fig. 2). Subjective image quality of all selected sequences was rated in favor of the group examined with the incubator. Figure 3 shows the average data of overall subjective image quality for both readers. The icMRI group showed a higher average diagnostic image quality than the ccMRI group, with scores rising from 3.5 to 4.3 points on the Likert scale, equivalent to an improvement from 69% to 86%. Motion artifacts occurred in 31% of ccMRI images but only in 14% of icMRI images. According to the quadratic weighted Cohen’s kappa test, the interobserver agreement statistics indicate substantial to almost perfect agreement. Detailed agreement tables for overall image quality are presented in Table 3. Figure 4 shows examples of image quality in different patients from the icMRI group, compared to the ccMRI group. Additionally, the analysis of the icMRI group based on the MRI devices used demonstrated that this did not lead to any changes in image quality (Table 4) within the icMRI group.

**Table 3:** Image quality scores for both groups by Reader 1 (R1) and Reader 2 (R2) using a 5-point Likert scale with 5 being the best, including quadratic weighted Cohen’s kappa (ϰ)

	icMRI group			ccMRI group		
	R1	R2	ϰ	R1	R2	ϰ
T1 transversal	4.0 ± 0.7	4.0 ± 0.8	0.89	3.0 ± 0.8	3.3 ± 0.9	0.67
T1 sagittal	3.8 ± 0.9	3.9 ± 0.8	0.89	3.1 ± 0.8	3.4 ± 0.9	0.73
T2 transversal	4.7 ± 0.6	4.7 ± 0.6	0.80	3.9 ± 1.1	4.0 ± 0.8	0.91
T2 sagittal	4.5 ± 0.9	4.1 ± 0.8	0.88	3.7 ± 0.6	3.9 ± 0.8	0.66
Dual TSE 1	4.3 ± 0.9	4.1 ± 0.8	0.77	4.2 ± 0.9	4.1 ± 0.9	0.92
Dual TSE 2	4.5 ± 0.8	4.6 ± 0.8	0.90	4.3 ± 1.1	4.3 ± 0.9	0.69
SWI	4.4 ± 0.7	4.4 ± 0.7	0.70	4.0 ± 0.8	3.8 ± 0.9	0.72
DWI	4.5 ± 0.6	4.3 ± 0.7	0.62	3.7 ± 0.8	3.6 ± 0.8	0.64

*cMRI* cerebral magnetic resonance imaging, *icMRI group* patient group undergoing cMRI with a recently developed 16-channel radiofrequency receiving head coil MR-compatible incubator, *ccMRI* *group* patient group undergoing conventional cMRI, *DWI* diffusion-weighted imaging, *SWI* susceptibility-weighted imaging, *TSE* Turbo spin echo

**Table 4:** Image quality parameters of the icMRI group for the two types of MRI scanners used

	1.5-T Achieva SmartPath to dStream					1.5-T Ingenia					*p*	*p*
	R1	R2	ϰ	SNRb	SNRc	R1	R2	ϰ	SNRb	SNRc	SNRb	SNRc
T1 transversal	4.0 ± 0.7	4.0 ± 0.8	0.84	520 ± 210	460 ± 200	3.0 ± 0.8	3.3 ± 0.9	0.67	460 ± 100	430 ± 90	*p* = 0.3	*p* = 0.9
T1 sagittal	3.8 ± 0.9	3.9 ± 0.8	0.89	440 ± 80	420 ± 100	3.1 ± 0.8	3.4 ± 0.9	0.73	500 ± 190	520 ± 230	*p* = 0.4	*p* = 0.08
T2 transversal	4.7 ± 0.6	4.7 ± 0.6	0.80	1200 ± 400	1500 ± 500	3.9 ± 1.1	4.0 ± 0.8	0.91	1400 ± 500	1800 ± 500	*p* = 0.8	*p* = 0.5
T2 sagittal	4.5 ± 0.9	4.1 ± 0.8	0.88	1600 ± 500	1900 ± 600	3.7 ± 0.6	3.9 ± 0.8	0.66	1700 ± 500	2000 ± 600	*p* = 0.3	*p* = 0.3
Dual TSE 1	4.3 ± 0.9	4.1 ± 0 8	0.77	1140 ± 160	1220 ± 160	4.2 ± 0.9	4.1 ± 0.9	0.92	1280 ± 260	1370 ± 260	*p* = 0.2	*p* = 0.07
Dual TSE 2	4.5 ± 0.8	4.6 ± 0.8	0.90	610 ± 120	790 ± 130	4.3 ± 1.1	4.3 ± 0.9	0.69	660 ± 110	860 ± 120	*p* = 0.3	*p* = 0.09
SWI	4.4 ± 0.7	4.4 ± 0.7	0.70	1420 ± 170	1380 ± 220	4.0 ± 0.8	3.8 ± 0.9	0.72	1470 ± 150	1470 ± 170	*p* = 0.4	*p* = 0.2
DWI	4.5 ± 0.6	4.3 ± 0.7	0.62	390 ± 70	350 ± 70	3.7 ± 0.8	3.6 ± 0.8	0.64	460 ± 190	470 ± 250	*p* = 0.1	*p* = 0.06

Image quality parameters: 5-point Likert scale for Reader 1 (R1) and Reader 2 (R2), quadratic weighted Cohen’s kappa (ϰ), SNRc, and SNRb

*cMRI* cerebral magnetic resonance imaging, *icMRI group* patient group undergoing cMRI with a recently developed MR-compatible incubator, *DWI* diffusion-weighted imaging, *SWI* susceptibility-weighted imaging, *SNRb* Signal-to-noise ratios in the basal ganglia, *SNRc* Signal-to-noise ratios in the parieto-occipital white matter, *TSE* Turbo spin echo

In both groups, 70% of the MRI examinations provided additional diagnostic information compared to the previously documented results of transfontanellar ultrasound. In the icMRI group, cMRI offered additional diagnostic value in 37 of 53 cases, whereas in the ccMRI group, such added value was observed in 35 of 50 cases.

### Analysis of anesthetic protocols

General anesthesia was administered to three patients in the icMRI group and ten patients in the ccMRI group (*p* = 0.03). All other patients underwent imaging with natural airway management, comprising 47 neonates in the icMRI group who required sedation and three who were examined without any anesthesia using the “feed-and-wrap” technique. In contrast, 40 patients in the ccMRI group required sedation, with none undergoing imaging without anesthesia (*p* = 0.3; *p* = 0.09), as detailed in Table 2.

## Discussion

This retrospective study included 103 consecutive term and preterm neonates who underwent clinically indicated cMRI. The neonates were categorized into two groups based on whether cMRI was carried out using ccMRI or a recently developed MR-compatible incubator with a 16-channel radiofrequency receiving head (icMRI). Both groups showed no significant differences in age, gender, or imaging indications. The main findings indicate that icMRI is associated with shorter examination times, improved diagnostic image quality, fewer motion artifacts, and a reduced need for general anesthesia. However, neonates in the icMRI group were significantly smaller with lower birth weight and length, but also at the time of examination. This difference likely reflects that our pediatricians from the neonatal intensive care unit were more inclined to request MRI scans when an incubator was available, as it allowed for improved monitoring of both term and preterm neonates.

Pediatric MRI often necessitates general anesthesia or sedation to ensure patient stillness during imaging. Previous research has established a direct correlation between anesthetic exposure and the duration of MRI scans [15]. The impacts and significance of general anesthesia, particularly in preterm and term neonates, must be carefully questioned and applied [16–18]. Given neonates’ vulnerability, reducing the need for anesthesia is crucial for minimizing potential risks and optimizing patient safety during imaging procedures. Our findings show that icMRI was associated with a reduced need for general anesthesia and a significantly shorter overall imaging time. These results suggest that icMRI contributes to making neonatal MRI both safer and more efficient.

Previous studies have confirmed the superior image quality achieved with MR-compatible incubators [7, 8, 19], emphasizing the critical importance of minimizing motion artifacts to enhance diagnostic accuracy. While earlier research has demonstrated the benefits of previous incubator models equipped with 8-channel radiofrequency head coils, such as improved feasibility and reduced motion artifacts [7], there are currently no published data on incubator systems featuring integrated 16-channel radiofrequency receiving head coils. Our findings address this gap by demonstrating that the use of an incubator system with a 16-channel head coil is associated with improved feasibility and significantly superior image quality compared to ccMRI. This reduces the need for repeat imaging and enables more accurate assessments of the neonatal brain. In line with previous studies, our findings further underscore the crucial diagnostic value of MRI-based cerebral imaging [3, 20–23].

Our study has several limitations. Firstly, this is not a prospective study with a fixed examination protocol. The examination of preterm or full-term infants is rare and should ideally be conducted within the framework of clinical routine, as these infants are fragile and should not be unnecessarily subjected to an MRI scan. Therefore, we opted for a retrospective study to include the largest possible variety of sequences. Except for the number of applications in the DWI sequences, no significant differences were observed between the two groups. This limitation highlights the necessity of further improving imaging protocols to ensure optimal benefit for pediatric patients. Unfortunately, due to the retrospective study design, it was not feasible to assess whether individual sequences required repetition or to determine the specific reasons for interruptions or prolonged examination times. Additionally, as part of the clinical routine, the icMRI examinations were conducted using two different MRI devices, which differed only in bore diameter. Furthermore, we were able to demonstrate that the different bore diameters of the two MRI devices did not result in a significant difference in image quality in the infants who were examined using an incubator. This was also to be expected, as the SNR is primarily influenced by the receiving coil, which was for the icMRI identical on both scanners, and less by the different diameters of the transmit coil, which is determined by the bore diameter. Another potential limitation of our retrospective study is the variation in imaging parameters between the icMRI and ccMRI groups in certain sequences, as shown in Table 1. For instance, the Dual TSE sequences differed in slice thickness and number of averages. These variations resulted from protocol optimizations enabled by the use of the incubator system with its integrated 16-channel radiofrequency head coil.

## Conclusion

Ensuring the safety of critically ill term and preterm neonates during MRI remains complex, requiring secure transportation to and from the MRI unit, maintenance of a stable environment during the procedure, and continuous monitoring of vital signs during imaging. The incubator system used in this study fulfills these requirements. Furthermore, its use was associated with improved feasibility and superior image quality across all evaluated sequences. In addition, the incubator also contributes to patient safety, as its use has been correlated with a reduced need for general anesthesia and increased procedural efficiency through shorter scan times in neonatal cerebral imaging compared to ccMRI.

## Abbreviations

ccMRI: Conventional cerebral magnetic resonance imaging
cMRI: Cerebral magnetic resonance imaging
DICOM: Digital imaging and communications in medicine
DWI: Diffusion-weighted imaging
icMRI: Cerebral MRI using a recently developed MR-compatible incubator
SNR: Signal-to-noise ratio
SNRb: Signal-to-noise ratios in the basal ganglia
SNRc: Signal-to-noise ratios in the parieto-occipital white matter
SWI: Susceptibility-weighted imaging
TSE: Turbo spin echo
